# Supplementary material for: NEOage clocks - epigenetic clocks to estimate post-menstrual and postnatal age in preterm infants
Source: Aging (Albany NY). 2021 Oct 16;13(20):23527–44. doi: 10.18632/aging.203637 (PMC8580352; doi:10.18632/aging.203637)
Supplement: Supplementary Tables 5-13 [file aging-13-203637-s005.pdf]

SUPPLEMENTARY TABLES

Supplementary Table 5. Top 20 pathways from GO pathway analysis for CpGs included in the 450k-PMA NEOage clock.

	ONTOLOGY	TERM	N	DE	P.DE	FDR
GO:0051240	BP	positive regulation of multicellular organismal process	1717	55.5	0.000101530604212178	1
GO:0007343	BP	egg activation	5	2	0.000488637355841379	1
GO:0071230	BP	cellular response to amino acid stimulus	63	7	0.000539308264807122	1
GO:0120162	BP	positive regulation of cold-induced thermogenesis	96	8	0.000645109263846585	1
GO:0005086	MF	ARF guanyl-nucleotide exchange factor activity	18	4	0.000719253481406075	1
GO:0032011	BP	ARF protein signal transduction	18	4	0.00124599435874616	1
GO:0032012	BP	regulation of ARF protein signal transduction	18	4	0.00124599435874616	1
GO:0043200	BP	response to amino acid	106	8	0.00126159591846291	1
GO:0031669	BP	cellular response to nutrient levels	223	11	0.00174701422017418	1
GO:0098772	MF	molecular function regulator	1668	45	0.00185581614546516	1
GO:0051239	BP	regulation of multicellular organismal process	3091	80	0.00185939492722176	1
GO:0002687	BP	positive regulation of leukocyte migration	120	7	0.00199912401286309	1
GO:0031668	BP	cellular response to extracellular stimulus	254	12	0.00200618606306946	1
GO:0071496	BP	cellular response to external stimulus	322	14	0.00209524302848006	1
GO:0051954	BP	positive regulation of amine transport	34	4	0.00234424561108088	1
GO:0045623	BP	negative regulation of T-helper cell differentiation	15	3	0.00247101470290018	1
GO:0106106	BP	cold-induced thermogenesis	140	9	0.00249643374976729	1
GO:0120161	BP	regulation of cold-induced thermogenesis	140	9	0.00249643374976729	1
GO:0042636	BP	negative regulation of hair cycle	5	2	0.00301157639678554	1
GO:0009966	BP	regulation of signal transduction	2976	75.33333333	0.00329481415897157	1

Supplementary Table 6. Top 20 pathways from KEGG pathway analysis for CpGs included in the 450k-PMA NEOage clock.

	Description	N	DE	P.DE	FDR
path:hsa04974	Protein digestion and absorption	92	7	0.0025432527716253	0.826463250084872
path:hsa04144	Endocytosis	242	12.5	0.00483311842154896	0.826463250084872
path:hsa04512	ECM-receptor interaction	86	6	0.0131031855183759	1
path:hsa04151	PI3K-Akt signaling pathway	331	13.5	0.0218884783349282	1
path:hsa00450	Selenocompound metabolism	17	2	0.0265841978233973	1
path:hsa04972	Pancreatic secretion	93	5	0.0269679372038782	1
path:hsa04971	Gastric acid secretion	75	5	0.0273248262064296	1
path:hsa04150	mTOR signaling pathway	151	7	0.0374334371410264	1
path:hsa04640	Hematopoietic cell lineage	89	4	0.0462122030513505	1
path:hsa04727	GABAergic synapse	84	5	0.0469447204065581	1
path:hsa03410	Base excision repair	32	2.5	0.0642826135929407	1
path:hsa04064	NF-kappa B signaling pathway	95	4	0.0737709112366848	1
path:hsa00500	Starch and sucrose metabolism	30	2	0.0746391342946885	1
path:hsa04510	Focal adhesion	193	8	0.0828799780172142	1
path:hsa04261	Adrenergic signaling in cardiomyocytes	144	6	0.0930959869894971	1
path:hsa05412	Arrhythmogenic right ventricular cardiomyopathy	74	4	0.106935318655637	1
path:hsa05200	Pathways in cancer	506	15	0.109313257881781	1
path:hsa00920	Sulfur metabolism	10	1	0.111928784033015	1
path:hsa04657	IL-17 signaling pathway	88	3	0.120766341082755	1
path:hsa03022	Basal transcription factors	41	2	0.122128583254498	1

Supplementary Table 7. Top 20 pathways from GO pathway analysis for CpGs included in the 450k-PNA NEOage clock.

	ONTOLOGY	TERM	N	DE	P.DE	FDR
GO:0042127	BP	regulation of cell population proliferation	1591	40	0.0000534190886299528	1
GO:0008283	BP	cell population proliferation	1897	44	0.000139796711649994	1
GO:0008285	BP	negative regulation of cell population proliferation	715	23	0.000144783294410145	1
GO:0090191	BP	negative regulation of branching involved in ureteric bud morphogenesis	2	2	0.000224072825185314	1
GO:2001252	BP	positive regulation of chromosome organization	164	9.5	0.000246284844795411	1
GO:0031616	CC	spindle pole centrosome	14	3	0.000330509425042432	1
GO:0033044	BP	regulation of chromosome organization	323	12.5	0.000664900082049856	1
GO:0045843	BP	negative regulation of striated muscle tissue development	55	5	0.000970314025106637	1
GO:1901187	BP	regulation of ephrin receptor signaling pathway	2	2	0.000971984410108729	1
GO:0048635	BP	negative regulation of muscle organ development	56	5	0.00108076577962778	1
GO:0097028	BP	dendritic cell differentiation	38	4	0.00125990537999378	1
GO:0060021	BP	roof of mouth development	88	7	0.00141641819931624	1
GO:1901862	BP	negative regulation of muscle tissue development	58	5	0.00153736679951646	1
GO:0030513	BP	positive regulation of BMP signaling pathway	32	4	0.00161955302866369	1
GO:0016202	BP	regulation of striated muscle tissue development	143	8	0.00163674469616389	1
GO:0048385	BP	regulation of retinoic acid receptor signaling pathway	16	3	0.00165543877096897	1
GO:0045082	BP	positive regulation of interleukin-10 biosynthetic process	4	2	0.00168336883391311	1
GO:0070534	BP	protein K63-linked ubiquitination	49	4	0.00175553877998935	1
GO:0048634	BP	regulation of muscle organ development	147	8	0.0019218112555276	1
GO:1901861	BP	regulation of muscle tissue development	146	8	0.00194065212296168	1

Supplementary Table 8. Top 20 pathways from KEGG pathway analysis for CpGs included in the 450k-PNA NEOage clock.

	Description	N	DE	P.DE	FDR
path:hsa04913	Ovarian steroidogenesis	50	3	0.0305045691205889	1
path:hsa04911	Insulin secretion	83	4	0.0580354487974986	1
path:hsa00920	Sulfur metabolism	10	1	0.0635494328977979	1
path:hsa00330	Arginine and proline metabolism	46	2	0.0660623210263602	1
path:hsa04664	Fc epsilon RI signaling pathway	66	3	0.0689829305658202	1
path:hsa00250	Alanine, aspartate and glutamate metabolism	36	2	0.0691860293452126	1
path:hsa04120	Ubiquitin mediated proteolysis	132	4	0.0765639489819126	1
path:hsa04261	Adrenergic signaling in cardiomyocytes	144	5	0.0797431390863962	1
path:hsa04020	Calcium signaling pathway	228	7	0.0802610307505027	1
path:hsa01100	Metabolic pathways	1400	21.5	0.0827951900967627	1
path:hsa00520	Amino sugar and nucleotide sugar metabolism	47	2	0.0837183667094073	1
path:hsa05221	Acute myeloid leukemia	64	3	0.0853382746769124	1
path:hsa04925	Aldosterone synthesis and secretion	95	4	0.0941511750167515	1
path:hsa04962	Vasopressin-regulated water reabsorption	43	2	0.0958227630574678	1
path:hsa00590	Arachidonic acid metabolism	61	2	0.0990080964986279	1
path:hsa04927	Cortisol synthesis and secretion	63	3	0.103475903215217	1
path:hsa05110	Vibrio cholerae infection	49	2	0.139903479319178	1
path:hsa04610	Complement and coagulation cascades	81	2	0.144076070674707	1
path:hsa00910	Nitrogen metabolism	15	1	0.144600617616157	1
path:hsa04725	Cholinergic synapse	111	4	0.144777378086761	1

Supplementary Table 9. Top 20 pathways from GO pathway analysis for CpGs included in the EPIC-PMA NEOage clock.

	ONTOLOGY	TERM	N	DE	P.DE	FDR
GO:0060090	MF	molecular adaptor activity	244	19.5	0.0000261984905881029	0.299312820997077
GO:0030674	MF	protein-macromolecule adaptor activity	204	17.5	0.0000264948943079647	0.299312820997077
GO:0009653	BP	anatomical structure morphogenesis	2628	93.3333333333333	0.00036075400639525	1
GO:0061061	BP	muscle structure development	649	30	0.000817243519837221	1
GO:0071149	CC	TEAD-2-YAP complex	2	2	0.000829613936436568	1
GO:0060187	CC	cell pole	2	2	0.00124016610690871	1
GO:0043005	CC	neuron projection	1245	51	0.00135112812526085	1
GO:0001725	CC	stress fiber	66	8	0.00149094428870597	1
GO:0097517	CC	contractile actin filament bundle	66	8	0.00149094428870597	1
GO:2000096	BP	positive regulation of Wnt signaling pathway, planar cell polarity pathway	8	3	0.00164092892480337	1
GO:0034330	BP	cell junction organization	629	32	0.00190548862751066	1
GO:0019215	MF	intermediate filament binding	14	3	0.00192713729059663	1
GO:0032432	CC	actin filament bundle	72	8	0.00213251244179354	1
GO:0032289	BP	central nervous system myelin formation	3	2	0.00247281453328891	1
GO:0090258	BP	negative regulation of mitochondrial fission	3	2	0.0026011582719471	1
GO:0003012	BP	muscle system process	457	21.8333333333333	0.00276093280594124	1
GO:0044297	CC	cell body	537	25.5	0.0030219067814788	1
GO:1904636	BP	response to ionomycin	4	2	0.00306956109657764	1
GO:1904637	BP	cellular response to ionomycin	4	2	0.00306956109657764	1
GO:0071936	MF	coreceptor activity involved in Wnt signaling pathway	8	3	0.00306994667197237	1

Supplementary Table 10. Top 20 pathways from KEGG pathway analysis for CpGs included in the EPIC-PMA NEOage clock.

	Description	N	DE	P.DE	FDR
path:hsa04310	Wnt signaling pathway	160	11	0.00563804820140634	1
path:hsa03022	Basal transcription factors	41	3	0.0382585766688834	1
path:hsa04150	mTOR signaling pathway	151	8	0.0409548512453372	1
path:hsa04514	Cell adhesion molecules	133	7	0.055839619373039	1
path:hsa04916	Melanogenesis	101	6	0.0575374173755753	1
path:hsa04750	Inflammatory mediator regulation of TRP channels	97	6.5	0.0678487404242832	1
path:hsa04971	Gastric acid secretion	75	5	0.0701572968381165	1
path:hsa00780	Biotin metabolism	3	1	0.0745147803124464	1
path:hsa04744	Phototransduction	27	2	0.080805733488801	1
path:hsa00630	Glyoxylate and dicarboxylate metabolism	30	2	0.0970358200187176	1
path:hsa04625	C-type lectin receptor signaling pathway	103	5	0.0984908711417847	1
path:hsa05146	Amoebiasis	98	5	0.0985952263276953	1
path:hsa05205	Proteoglycans in cancer	199	9	0.0989759531598264	1
path:hsa00515	Mannose type O-glycan biosynthesis	23	2	0.0996538362015267	1
path:hsa03410	Base excision repair	32	2.5	0.101507997479545	1
path:hsa04144	Endocytosis	244	10	0.102761294195724	1
path:hsa05031	Amphetamine addiction	66	4	0.107648802928742	1
path:hsa04152	AMPK signaling pathway	117	6	0.109755427511973	1
path:hsa04080	Neuroactive ligand-receptor interaction	320	9.16666666666667	0.125111384608538	1
path:hsa04070	Phosphatidylinositol signaling system	92	5	0.12605022083487	1

Supplementary Table 11. Top 20 pathways from GO pathway analysis for CpGs included in the EPIC-PNA NEOage clock.

	ONTOLOGY	TERM	N	DE	P.DE	FDR
GO:0110111	BP	negative regulation of animal organ morphogenesis	33	7	0.0000150081855686758	0.339094944738661
GO:1905331	BP	negative regulation of morphogenesis of an epithelium	16	5	0.0000864716932197235	0.951641205696613
GO:0060686	BP	negative regulation of prostatic bud formation	4	3	0.000172823666721358	0.951641205696613
GO:0030510	BP	regulation of BMP signaling pathway	88	9	0.000218810440561404	0.951641205696613
GO:0090191	BP	negative regulation of branching involved in ureteric bud morphogenesis	2	2	0.000250881472848229	0.951641205696613
GO:0048645	BP	animal organ formation	63	8.5	0.000320898779391021	0.951641205696613
GO:0060685	BP	regulation of prostatic bud formation	5	3	0.000364936681518837	0.951641205696613
GO:0060688	BP	regulation of morphogenesis of a branching structure	53	7	0.000371355157976279	0.951641205696613
GO:0090192	BP	regulation of glomerulus development	14	4	0.000382316795422211	0.951641205696613
GO:0072283	BP	metanephric renal vesicle morphogenesis	14	4	0.000504258477605173	0.951641205696613
GO:0030509	BP	BMP signaling pathway	146	11	0.000555551138375544	0.951641205696613
GO:0032675	BP	regulation of interleukin-6 production	143	9	0.000582041777636664	0.951641205696613
GO:0005402	MF	carbohydrate:cation symporter activity	19	4	0.000606490301815559	0.951641205696613
GO:0090185	BP	negative regulation of kidney development	16	4	0.000644477997689534	0.951641205696613
GO:0047045	MF	testosterone 17-beta-dehydrogenase (NADP+) activity	4	2	0.000704248067798324	0.951641205696613
GO:2000343	BP	positive regulation of chemokine (C-X-C motif) ligand 2 production	9	3	0.000725385020377788	0.951641205696613
GO:0032755	BP	positive regulation of interleukin-6 production	91	7	0.000751360618487751	0.951641205696613
GO:0030432	BP	peristalsis	10	3	0.000839410257077136	0.951641205696613
GO:0072006	BP	nephron development	137	11	0.000849343536609647	0.951641205696613
GO:0030513	BP	positive regulation of BMP signaling pathway	32	5	0.000906591713866725	0.951641205696613

Supplementary Table 12. Top 20 pathways from KEGG pathway analysis for CpGs included in the EPIC-PNA NEOage clock.

	Description	N	DE	P.DE	FDR
path:hsa05110	Vibrio cholerae infection	49	5	0.0044303057296943	1
path:hsa04961	Endocrine and other factor-regulated calcium reabsorption	51	5	0.0130902253578704	1
path:hsa04925	Aldosterone synthesis and secretion	95	7	0.0177978978310381	1
path:hsa04920	Adipocytokine signaling pathway	66	5	0.022338795028672	1
path:hsa04650	Natural killer cell mediated cytotoxicity	118	6.5	0.0315357173452436	1
path:hsa04217	Necroptosis	147	6	0.0396709182209861	1
path:hsa04261	Adrenergic signaling in cardiomyocytes	144	8	0.0399713503271485	1
path:hsa00980	Metabolism of xenobiotics by cytochrome P450	74	3	0.0439232520019814	1
path:hsa04970	Salivary secretion	86	5	0.0473923210414602	1
path:hsa00511	Other glycan degradation	18	2	0.0566603395732447	1
path:hsa05217	Basal cell carcinoma	63	4	0.062286340491488	1
path:hsa05412	Arrhythmogenic right ventricular cardiomyopathy	74	5	0.0666165663705422	1
path:hsa04020	Calcium signaling pathway	228	10.5	0.0733984670436083	1
path:hsa04060	Cytokine-cytokine receptor interaction	278	7	0.0823487304128945	1
path:hsa04978	Mineral absorption	54	3	0.0861725768552945	1
path:hsa04727	GABAergic synapse	84	5	0.0868375944591549	1
path:hsa04061	Viral protein interaction with cytokine and cytokine receptor	97	3.5	0.0971045985176263	1
path:hsa04935	Growth hormone synthesis, secretion and action	116	6	0.103216705086696	1
path:hsa05414	Dilated cardiomyopathy	92	5	0.10503579562344	1
path:hsa04066	HIF-1 signaling pathway	105	5	0.111209287977195	1

Supplementary Table 13. Annotation of the 20 common CpGs of the NEOage clocks.

Name	chr	pos	UCSC_RefGene_Name
cg05394010	chr16	2546596	TBC1D24
cg04777726	chr19	49340489	PLEKHA4;PLEKHA4;HSD17B14
cg24541835	chr1	12651540	DHRS3
cg05265234	chr22	38884016	DDX17;DDX17;DDX17;DDX17;DDX17;DDX17
cg21219851	chr17	78898189	RPTOR;RPTOR
cg00049440	chr9	73026643	KLF9
cg01454951	chr3	71730677	EIF4E3;EIF4E3;EIF4E3;EIF4E3
cg21664351	chr11	19841423	NAV2;NAV2;NAV2
cg12266861	chr1	35449720	
cg07318287	chr1	154377429	IL6R;IL6R
cg04862002	chr17	9074365	NTN1
cg00465247	chr13	50703477	
cg01138164	chr7	96648447	
cg13942103	chr1	111177829	
cg21135560	chr8	144946659	EPPK1
cg01916724	chr3	51975220	PARP3;PARP3;RRP9
cg05624226	chr5	180325954	BTNL8;BTNL8;BTNL8;BTNL8;BTNL8
cg13624964	chr14	86088696	FLRT2
cg17995197	chr2	26408167	FAM59B
cg06002476	chr3	8617065	
