# Supplementary material for: NEOage clocks - epigenetic clocks to estimate post-menstrual and postnatal age in preterm infants
Source: Aging (Albany NY). 2021 Oct 16;13(20):23527–44. doi: 10.18632/aging.203637 (PMC8580352; doi:10.18632/aging.203637)
Supplement: Supplementary Code 1 [file aging-13-203637-s006.docx]

**Supplementary Code 1. R code example to calculate DNAm age using NEOage clocks.**

library(minfi) # logit2

# logit transformed DNA methylation data (datM; columns: samples, rows: CpGs)

datM = logit2(datM)

# PMA EPIC

## reading in clock CpGs with coefficients

NEOage_EPIC_PMA_clock=read.csv("05-13-2021_PMA-EPIC.csv", header = T)

## subset and transform DNAm data to selected CpGs

dataClock = data.frame(t(datM[NEOage_EPIC_PMA_clock$ID[-1],]))

## calculate NEOage

NEOage_EPIC_PMA = as.numeric(NEOage_EPIC_PMA_clock[1,2] + as.numeric(as.matrix(dataClock) %*% as.numeric(NEOage_EPIC_PMA_clock$coef[-1])))

# PMA 450k

## reading in clock CpGs with coefficients

NEOage_450k_PMA_clock=read.csv("05-13-2021_PMA-450k.csv", header = T)

## subset and transform DNAm data to selected CpGs

dataClock = data.frame(t(datM[NEOage_450k_PMA_clock$ID[-1],]))

## calculate NEOage

NEOage_450k_PMA = as.numeric(NEOage_450k_PMA_clock[1,2] + as.numeric(as.matrix(dataClock) %*% as.numeric(NEOage_450k_PMA_clock$coef[-1])))

# PNA EPIC

## reading in clock CpGs with coefficients

NEOage_EPIC_PNA_clock=read.csv("05-13-2021_PNA-EPIC.csv", header = T)

## subset and transform DNAm data to selected CpGs

dataClock = data.frame(t(datM[NEOage_EPIC_PNA_clock$ID[-1],]))

## calculate NEOage

NEOage_EPIC_PNA = as.numeric(NEOage_EPIC_PNA_clock[1,2] + as.numeric(as.matrix(dataClock) %*% as.numeric(NEOage_EPIC_PNA_clock$coef[-1])))

# PNA 450k

## reading in clock CpGs with coefficients

NEOage_450k_PNA_clock=read.csv("05-13-2021_PNA-450k.csv", header = T)

## subset and transform DNAm data to selected CpGs

dataClock = data.frame(t(datM[NEOage_450k_PNA_clock$ID[-1],]))

## calculate NEOage

NEOage_450k_PNA = as.numeric(NEOage_450k_PNA_clock[1,2] + as.numeric(as.matrix(dataClock) %*% as.numeric(NEOage_450k_PNA_clock$coef[-1])))
